# A review of the genus *Sinocymbachus* Strohecker & Chûjô with description of four new species (Coleoptera, Endomychidae)

**DOI:** 10.3897/zookeys.936.48577

**Published:** 2020-05-28

**Authors:** Ling-Xiao Chang, Wen-Xuan Bi, Guo-Dong Ren

**Affiliations:** 1 Science Research Department, Beijing Museum of Natural History, Beijing 100050, China Science Research Department, Beijing Museum of Natural History Beijing China; 2 Room 401, No. 2, Lane 155, Lianhua South Road, Shanghai 201100, China unaffiliated Shanghai China; 3 College of Life Sciences, Hebei University, Baoding 071002, China Hebei University Baoding China

**Keywords:** Coleoptera, Endomychidae, new species, taxonomy

## Abstract

This work presents a review of species of the Asian genus *Sinocymbachus* Strohecker & Chûjô, 1970. Four new species are described from China: *S. fanjingshanensis* Chang & Bi, **sp. nov.**, *S. longipennis* Chang & Bi, **sp. nov.**, *S. sinicus* Chang & Bi, **sp. nov.**, and *S. wangyinjiei* Chang & Bi, **sp. nov.***Cymbachus koreanus* Chûjô & Lee, 1993 is transferred to *Sinocymbachus* as *S. koreanus* (Chûjô & Lee) **comb. nov.***Sinocymbachus bimaculatus* (Pic, 1927) is reported for the first time from China. The male of *S. parvimaculatus* (Mader, 1938) is discovered and described for the first time. Illustration, diagnosis and distribution are provided for each species. Prior to the present study, *Sinocymbachus* included ten species. An updated key to the species of *Sinocymbachus* is given.

## Introduction

The genus *Sinocymbachus* is exclusively known in Southeast Asia and was established by Strohecker and Chûjô (1970) with *Engonius excisipes* Strohecker, 1943 from China (Sichuan) as the type species. It is a member of the largest endomychid subfamily Lycoperdininae, the monophyly of which was tested and confirmed by the phylogenetic analyses of morphological characters by Tomaszewska (2000, 2005). Robertson et al. (2015) presented a large-scale phylogenetic study for the Cucujoidea, using molecular evidence to rebuild the relationship tree of this superfamily and established a new superfamily, Coccinelloidea, with Endomychidae placed within it. This study further confirmed the monophyly of the subfamily Lycoperdininae and established its sister relationship with the subfamily Epipocinae (Robertson et al. 2015).

Tomaszewska (2005) recognised five generic groups among 38 genera of Lycoperdininae known at that time; she placed *Sinocymbachus* with eight other genera in the *Amphix*-group containing *Amphix* Laporte, 1840 (returned to *Corynomalus* Chevrolat (Bousquet 2004, Arriaga Varela et al. 2007, Shockley et al. 2009)), *Acinaces* Gerstaecker, 1858 (11 species), *Beccariola* Arrow, 1943 (31 species), *Dryadites* Frivaldszky, 1883 (8 species), *Cymbachus* Gerstaecker, 1857 (5 species), *Pseudindalmus* Arrow, 1920 (13 species), *Aphorista* Gorham, 1873 (3 species) and *Mycetina* Mulsant, 1846 (70 species) widely distributed in the Holarctic, Oriental, and Afrotropical regions. This group is supported by a larval synapomorphy: labrum with sinuate or multi-denticulate apical margin (Tomaszewska 2005).

Apart from *Engonius excisipes*, Strohecker and Chûjô (1970) transferred seven other species to *Sinocymbachus*: *Cymbachus humerosus* Mader, 1938, *C. parvimaculatus* Mader, 1938, *Engonius luteomaculatus* Pic, 1921, *E. angustefasciatus* Pic, 1940, *Amphisternus bimaculatus* Pic, 1927, *A. quadrimaculatus* Pic, 1927, and *A. quadriundulatus* Chûjô, 1938. Moreover, *Amphisternus quadrinotatus* Chûjô, 1938 was recognised as a synonym of *S. humerosus* (Mader, 1938) by the same authors, and *Sinocymbachus politus* (Taiwan) and *S. decorus* (Yunnan) were described as new species (Strohecker and Chûjô 1970).

Prior to the present study, *Sinocymbachus* included ten species (Shockley et al. 2009): *S. angustefasciatus*, *S. bimaculatus*, *S. decorus*, *S. excisipes*, *S. humerosus*, *S. luteomaculatus*, *S. parvimaculatus*, *S. politus*, *S. quadrimaculatus* and *S. quadriundulatus*. During the examination of Endomychidae collected in China, four new species were recognised and are described here. An updated key to species of *Sinocymbachus* is given.

## Materials and methods

Type specimens of the new species described here and examined specimens are deposited in the following institutions and private collections:

**CBWX** Collection of Wen-Xuan Bi, Shanghai, China

**CCCC** Collection of Chang-Chin Chen, Tianjin, China

**CCLX** Collection of Ling-Xiao Chang, Beijing, China

**IZCAS** Chinese Academy of Sciences, Institute of Zoology, Beijing, China

**MHBU** Museum of Heibei University, Baoding, China

**SHNU** Shanghai Normal University, China, Shanghai

The specimens were examined, dissected, and measured using a Olympus SZX10 dissecting microscope. The measurements are standardised as follows: body length from the apical margin of the clypeus to the apex of the elytra; body width across both elytra at widest part; pronotal length from anterior angle to posterior margin; elytral length along the suture, including the scutellum. After observation, the dissected parts were mounted on the same card with the specimen. The abdomen was boiled in 10% NaOH solution, cleaned, and the aedeagus was dissected in distilled water. Habitus photographs were taken using a Canon EOS 5D III SLR camera and Canon MP-E 65 mm macro lens, and an Olympus OM-D E-M1 camera and Olympus ED 60 mm macro lens. Photographs of male genitalia and aedeagi were taken using a Canon EOS 5D III SLR camera and Canon MP-E 6 5mm macro lens. All photographs were refined in Adobe Photoshop CC 2015.

## Taxonomy

### Sinocymbachus

Taxon classificationAnimaliaColeoptera

Strohecker & Chûjô

677897FE-1396-5F88-93AD-8C4B09A574EC

Sinocymbachus Strohecker & Chûjô, 1970: 511.

#### Type species.

*Engonius excisipes* Strohecker, 1943.

#### Diagnosis.

The species of *Sinocymbachus* appear to be closely related to *Cymbachus*. However, *Sinocymbachus* can be distinguished from *Cymbachus* in having the body more elongate in most cases (except *S. parvimaculatus* and *S. sinicus* sp. nov.); intercoxal process of mesoventrite distinctly longer than wide (except *S. parvimaculatus* and *S. sinicus* sp. nov.), and with median ridge or tubercle at base; mesotibiae sexually dimorphic, toothed and excised on inner edge in male, straight in female (modified based on Tomaszewska 2005).

#### Remarks.

There is distinct sexual dimorphism in the species of the genus *Sinocymbachus*. The species of the genus *Sinocymbachus* bear distinct characters of sexual dimorphism. The mesotibiae of males usually have variously developed teeth and excisions. Besides, median lobe of the aedeagus is usually less complicated apically in the species with teeth and excisions symmetric on mesotibiae while it is more complicated in the species with teeth and excisions placed asymmetrically on tibiae and usually have a wide concavity on the ventral side of antenna. However, there are also exceptions. For example, the median lobe is complicated in the males of *S. excisipes* with symmetric teeth and excisions on mesotibiae while there is a wide concavity on the ventral surface of antenna in the males of *S. humerosus* with symmetrically placed teeth and excisions on mesotibiae.

### Sinocymbachus fanjingshanensis

Taxon classificationAnimaliaColeoptera

Chang & Bi sp. nov.

4FBE5B17-A1BC-5C72-8582-9177BDC1C7AB

http://zoobank.org/0022ECFA-7F83-4A4F-A3AC-57CEFDAB78F8

**Figure 1.**
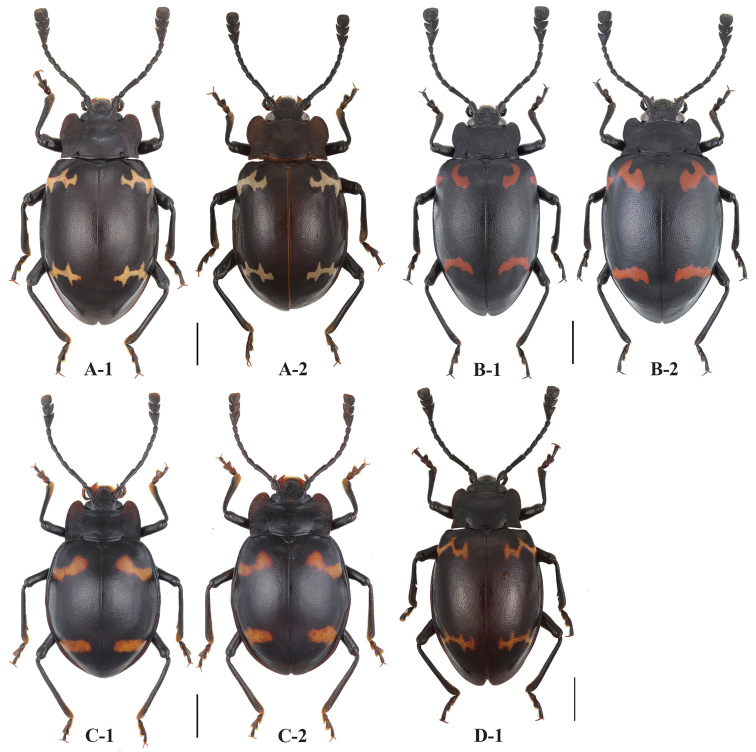
Habitus of *Sinocymbachus* spp. nov. **A***S. fanjingshanensis* sp. nov. **B***S. longipennis* sp. nov. **C***S. sinicus* sp. nov. **D***S. wangyinjiei* sp. nov. **1** male **2** female. Scale bar: 2 mm.

**Figure 2.**
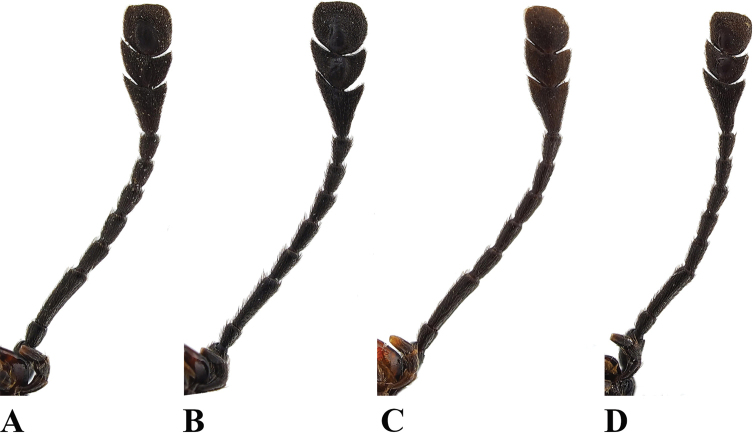
Left antenna of *Sinocymbachus* spp. nov. (ventral view) **A***S. fanjingshanensis* sp. nov. **B***S. longipennis* sp. nov. **C***S. sinicus* sp. nov. **D***S. wangyinjiei* sp. nov.

**Figure 3.**
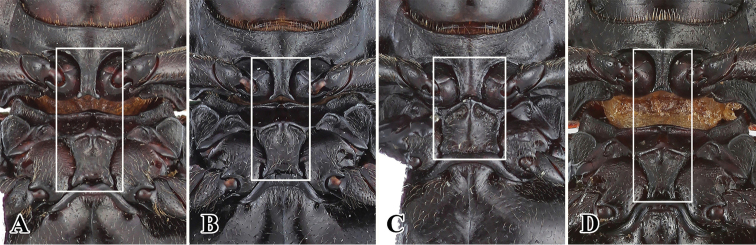
Prosternal and mesoventral process of *Sinocymbachus* spp. nov. **A***S. fanjingshanensis* sp. nov. **B***S. longipennis* sp. nov. **C***S. sinicus* sp. nov. **D***S. wangyinjiei* sp. nov.

**Figure 4.**
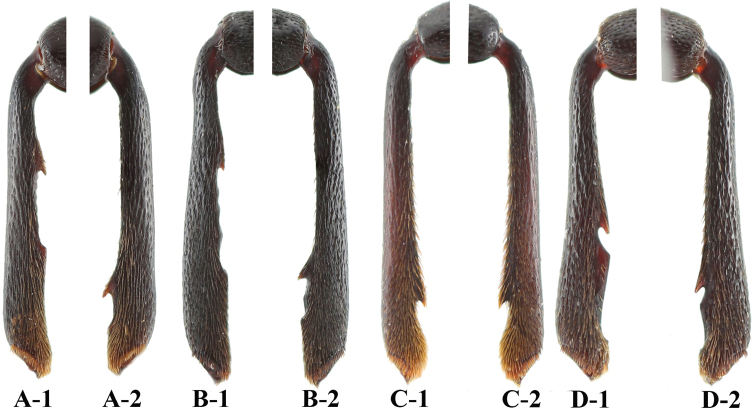
Male mesotibia of *Sinocymbachus* spp. nov. **A***S. fanjingshanensis* sp. nov. **B***S. longipennis* sp. nov. **C***S. sinicus* sp. nov. **D***S. wangyinjiei* sp. nov. **1** left **2** right.

**Figure 5.**
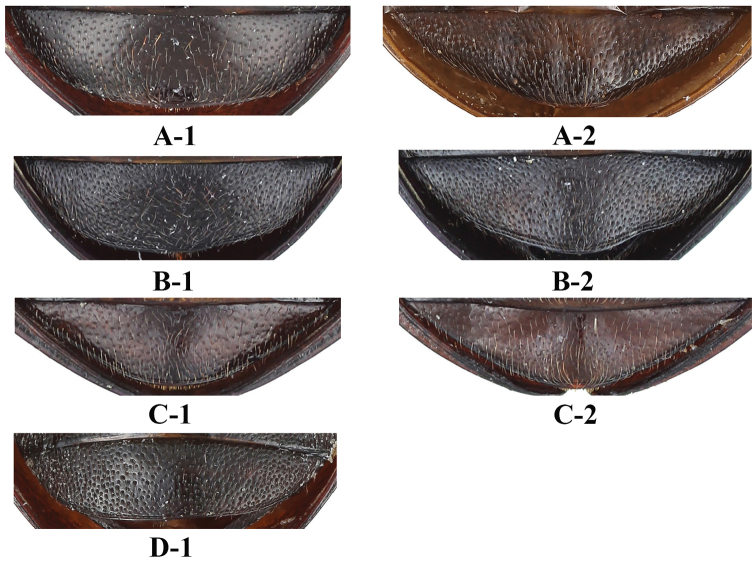
Male ventrite V of abdomen of *Sinocymbachus* spp. nov. **A***S. fanjingshanensis* sp. nov. **B***S. longipennis* sp. nov. **C***S. sinicus* sp. nov. **D***S. wangyinjiei* sp. nov. **1** male **2** female.

**Figure 6.**
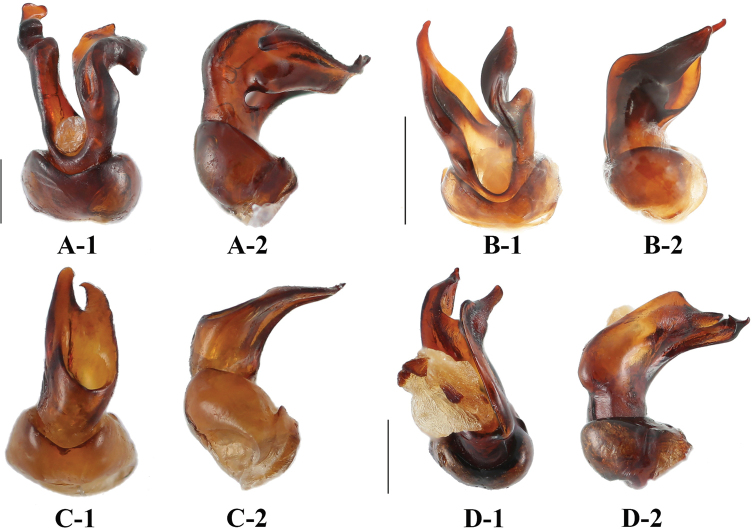
Aedeagus of *Sinocymbachus* spp. nov. **A***S. fanjingshanensis* sp. nov. **B***S. longipennis* sp. nov. **C***S. sinicus* sp. nov. **D***S. wangyinjiei* sp. nov. **1** ventral view **2** lateral view. Scale bar: 1 mm.

Figures 1A , 2A , 3A , 4A , 5A , 6A

#### Type material.

***Holotype*.** Male, China: Guizhou Province: Jiangkou, Fanjingshan, 1775 m, 23–27.VII.2016, Yu-Tang Wang leg. (SHNU); ***Paratypes*.** 1 male, 1 female, Jiangkou, Fanjingshan, 1775 m, 11.VII.2009, Wen-Hsin Lin leg. (CCCC).

#### Etymology.

The name refers to the type locality.

#### Diagnosis.

*Sinocymbachus fanjingshanensis* sp. nov. is most similar to *S. angustefasciatus*, *S. longipennis* sp. nov., *S. quadriundulatus* and *S. wangyinjiei* sp. nov. in appearance. However, *S. fanjingshanensis* sp. nov. differs from *S. angustefasciatus* by the shiny body (vs. body opaque); left mesotibia in male widely excised (vs. not excised); from *S. longipennis* sp. nov. by the body dark brown, shiny (vs. black, opaque); elytra oval with sides curved (vs. long oval, weakly curved); right mesotibia in male not excised (vs. with shallow and weakly undulate excision); from *S. quadriundulatus* by the body without cupreous sheen (vs. with cupreous sheen); left mesotibia in male widely excised (vs. not excised); from *S. wangyinjiei* sp. nov. by the mesoventral process with anterior and posterior margins nearly equally wide (vs. anterior margin much wider than posterior); mesotibia in male with shallow but broad excision (vs. with deep and narrow excision).

#### Description.

Length 9.7–10.9 mm. Body oval, approximately 1.9–2.0 times as long as wide; convex; shiny. Colour dark brown, shiny, with four irregular orange transverse maculae on the elytra.

***Head*.** Antenna (Fig. 2A) long, rather slender, extending to ca. 1/2 body length, with antennomeres 1–8 distinctly longer than wide; scape ca. 3.0 times as long as pedicel; pedicel wider than long; antennomere 3 nearly as long as 4 and 5 combined; antennomere 4 as long as 5; antennomeres 5–8 gradually shorter; club rather broad, moderately flat, loosely articulated; antennomeres 10 and 11 ventrally with wide concavity.

***Thorax*.** Pronotum 2.4–2.9 mm long, 3.5–3.8 mm wide; widest near 1/2 of pronotal length; rather coarsely and densely punctate; lateral and anterior margins narrowly bordered; anterior edge with large stridulatory membrane; sides undulate, deeply emarginate near basal 1/4; anterior angles produced, bluntly rounded; posterior angles acutely produced; disc weakly convex; median furrow absent; lateral sulci distinct, linear, extending to 1/3 of pronotal length; basal sulcus weakly sinuate, moderately deep. Prosternal process (Fig. 3A) moderately widely separating procoxae; sides almost parallel, forked apically, not extending beyond front coxae. Mesoventral process (Fig. 3A) nearly pentagonal, distinctly longer than wide; sides curved outwardly; disc with short median ridge near basal 1/4. Elytra 7.3–8.1 mm long, 5.2–5.5 mm wide; 2.8–3.0 times as long as pronotum; 1.4–1.5 times as wide as pronotum; punctations coarser and denser than those on pronotum; sides curved, widest near 1/2 of elytral length; converging from posterior 1/3 to apex; humeri weakly prominent. Each elytron with two transverse maculae: anterior macula behind humerus, nearly branch-shaped, with outer margin touching lateral margin of elytra, and inner margin distant from elytral suture; posterior macula located at apical 1/3, band-like, with anterior and posterior margins bidentate; outer margin distant from lateral margin of elytra, inner margin distant from elytral suture. Left mesotibia (Fig. 4A–1) in male with small sharp tooth near basal 1/4 on inner edge, then deeply excised to apical 1/3; right mesotibia (Fig. 4A–2) in male with small sharp tooth near apical 1/3 on inner edge. Pro- and metatibiae simple.

***Abdomen*.** Ventrite 1 almost as long as two subsequent ventrites combined; ventrites 2–4 subequal in length. Ventrite 5 with lateral margin gradually converging posteriorly; posterior margin broadly rounded medially in male (Fig. 5A–1); in female lateral margin strongly converging posteriorly; posterior margin acutely rounded medially (Fig. 5A–2). Aedeagus (Fig. 6A) short and stout, heavily sclerotised, curved. Median lobe very short, with wide branches. Tegmen basal, ring-shaped.

#### Distribution.

China: Guizhou.

### Sinocymbachus longipennis

Taxon classificationAnimaliaColeoptera

Chang & Bi sp. nov.

3854B435-558C-57AF-8060-A7D8690A748A

http://zoobank.org/CBB57831-D5E1-4CFF-A122-64FEF26CB4B6

Figures 1B , 2B , 3B , 4B , 5B , 6B

#### Type material.

***Holotype*.** Male, China: Yunnan Province: male, Gongshan, Dabadi, 2840 m, 14.VI.2015, Wen-Xuan Bi leg. (SHNU); ***Paratypes*.** 1 male, 1 female, same data as holotype (CBWX); 1 female, Gongshan, Sendang-Dabadi, 2840 m, 20.VI.2015, Wen-Xuan Bi leg. (CBWX); 1 male, Diqing, Weixi, Badi, Nanjieluo, 2921 m, 29.VI.2014, Xiao-Dong Yang leg. (CCCC); 5 males, Dayao County, Santai Township, Xiaobaicaoling, 29–30.V.2013, 2980 m Wen-Xuan Bi leg. (CBWX); 1 male, Binchuan County, Jizushan, 2400 m, 18.VII.2010, Liang Tang leg. (CBWX); Sichuan Province: Shimian County, alt. 2580 m, 5.VIII.2016, Ai-Min Shi, Yan-Hong Li leg. (MHBU); 9 males, Puge County, Luobinshan Cableway Station (downhill exit), alt. 2500 m, light trap, 9.VI.2012, Gan-Yan Yang leg. (IZCAS); 9 males, Puge County, Luobinshan, 2616 m, 9–10.VI.2012, Xiao-Dong Yang leg. (CCCC); 2 males, ditto except (CCLX); 1 male, Mianning County, Yele, 2800 m, 11.VIII.2005, Yi Ming leg. (CBWX); 12 males, Liziping, Shoubayan Power Station, alt. 2360 m, light trap, 28.9351N, 102.2468E, 22.VI.2012, Gan-Yan Yang leg. (IZCAS); Xizang Province: 1 female, Chawalong, Mengzhacun, Zhahazu, 2920 m, 3.VIII.2015, Wen-Xuan Bi leg. (CBWX).

#### Etymology.

The name refers to the distinctly elongate elytra, especially in male.

#### Diagnosis.

*Sinocymbachus longipennis* sp. nov. is most similar to *S. angustefasciatus*, *S. fanjingshanensis* sp. nov., *S. quadriundulatus* and *S. wangyinjiei* sp. nov. in external appearance. However, the distinctly more elongate elytra (especially in males) with nearly parallel sides can separate it from all these similar species. In addition, *S. longipennis* sp. nov. differs from *S. angustefasciatus* by having the elytra widest in the middle length (vs. behind middle); left mesotibia in male widely excised (vs. not widely excised); from *S. fanjingshanensis* sp. nov. by the body black and opaque (vs. dark brown, shiny); right mesotibia in male with shallow and weakly undulate excision (vs. not excised); from *S. quadriundulatus* by the body without cupreous sheen (vs. with cupreous sheen); left mesotibia in male widely excised (vs. not widely excised); from *S. wangyinjiei* sp. nov. by the body black and opaque (vs. dark brown, shiny); mesotibia in male with shallow and nearly straight excision (vs. with deeply U-shaped excision).

#### Description.

Length 9.5–10.6 mm. Body long and oval, 1.8 times as long as wide; convex; shiny. Colour black with four orange irregular maculae on elytra, narrow and transverse.

***Head*.** Antenna (Fig. 2B) long, rather stout, extending to ca. 1/2 body length, with antennomeres 1–8 distinctly longer than wide; scape approximately 3.5 times as long as pedicel; pedicel wider than long; antennomere 3 longer than 4 and 5 combined; antennomere 4 as long as 5; antennomeres 5–8 gradually shorter; club very broad, approximately 4.0 times as wide as antennomere 8, moderately flat, loose; antennomeres 10 and 11 ventrally with wide concavity.

***Thorax*.** Pronotum 2.3–2.8 mm long, 3.2–3.5 mm wide; widest at base; coarsely and rather densely punctate; lateral and anterior margins narrowly bordered; anterior edge with moderately large stridulatory membrane; sides undulate, deeply emarginate near basal 1/3 length; anterior angles produced, bluntly rounded; posterior angles acutely produced; disc weakly convex; median furrow absent; lateral sulci distinct, linear, extending to 1/3 of pronotal length; basal sulcus weakly sinuate, moderately deep. Prosternal process (Fig. 3B) moderately widely separating procoxae; sides curved outwardly, forked apically, not extending beyond front coxae. Mesoventral process (Fig. 3B) nearly pentagonal, with sides curved outwardly; disc distinctly ridged. Elytra 7.7–8.2 mm long, 5.2–5.8 mm wide; 2.9–3.3 times as long as pronotum; 1.6–1.7 times as wide as pronotum; punctations coarse and denser than those on pronotum; sides weakly curved, widest near 1/2 length of elytron; strongly converging from posterior 1/3 to apex; humeri weakly prominent. Each elytron with two transverse maculae: anterior elytral macula located on humerus, nearly W-shaped, in some specimens separated into two parts; outer margin almost touching lateral margin of elytra, inner margin distant from elytral suture; posterior macula located at apical 1/3, nearly band-shaped; outer margin distant from lateral margin of elytra, inner margin distant from elytral suture. Left mesotibia (Fig. 4B–1) in male with small sharp tooth near basal 1/3 on inner edge, then deeply excised to apical 1/3; right mesotibia (Fig. 4B–2) in male with small sharp tooth near apical 1/3 on inner edge. Pro- and metatibiae simple.

***Abdomen*.** Ventrite 1 almost as long as subsequent two ventrites combined; ventrites 2–4 subequal in length. Ventrite 5 with lateral margin gradually converging posteriorly; posterior margin broadly rounded in male (Fig. 5B–1); in female lateral margin strongly converging posteriorly and acutely rounded (Fig. 5B–2). Aedeagus (Fig. 6B) short and stout, heavily sclerotised, curved. Median lobe very short, with two large finger-like branches. Tegmen basal, ring-shaped.

#### Distribution.

China: Yunnan and Sichuan.

### Sinocymbachus sinicus

Taxon classificationAnimaliaColeoptera

Chang & Bi sp. nov.

9022353A-653D-52A7-B260-67139CF72882

http://zoobank.org/CAB67E33-813E-4C10-81A8-92E7A029B91E

Figures 1C , 2C , 3C , 4C , 5C , 6C

#### Type material.

***Holotype*.** Male, China: Xizang Province: Motuo, Baricun, 1700 m, 7.VIII.2014, Wen-Xuan Bi leg. (SHEM); ***Paratypes*.** 1 male, 1 female same data as holotype (CCLX); 1 male ditto except (CBWX); 1 female, Motuo, 1900 m, 20.VIII.2013, Wen-Xuan Bi leg. (CBWX); 2 females, Xizang, Linzhi, Motuo, 1559 m, 2016.VIII.5, Xiao-Dong Yang leg. (CCCC); 1 male, 1 female, Linzhi, Motuo, 1526 m, 2016.VIII.21, Xiao-Dong Yang leg. (CCCC); 1 male, ditto except 2016.VIII.23 (CCCC); 1 male, Yunnan Province: Gongshan, Dulongjiang, Maku, 1250 m, Wen-Xuan Bi leg. (CBWX).

#### Etymology.

The name is dedicated to our country which has created good research environment for us.

#### Diagnosis.

*Sinocymbachus sinicus* can be separated from all its congeners by having the body short oval and each elytron with two transverse maculae, more regular without distinct projections.

#### Description.

Length 8.9–9.1 mm. Body short oval, approximately 1.3 times as long as wide; moderately convex; smooth. Colour black with four orange irregular transverse maculae on elytra.

***Head*.** Antenna (Fig. 2C) long and slender, extending to approximately 1/2 body length, with antennomeres 1–8 distinctly longer than wide; scape approximately 4.0 times as long as pedicel; antennomere 3 longer than 4–5 combined; antennomere 4 nearly as long as antennomere 5; antennomeres 5–8 gradually shorter; club broad, approximately 2.5 times as wide as antennomere 8, moderately flat, loosely articulated.

***Thorax*.** Pronotum 2.3–2.4 mm long, 3.5–3.9 mm wide; widest near 1/2 of pronotal length; coarsely and rather densely punctate; lateral and anterior margins narrowly bordered; anterior edge with moderately large stridulatory membrane; sides nearly parallel; anterior angles produced, bluntly rounded; posterior angles moderately acutely produced; disc weakly convex; two round raised areas laterally; inflexed laterally; median furrow absent; lateral sulci very short and deep, in form of triangular dent; basal sulcus weakly curved, moderately deep. Prosternal process (Fig. 3C) comparatively widely separating procoxae; sides curved outwardly near apex, forked apically, not extending beyond front coxae. Mesoventral process (Fig. 3C) nearly pentagonal, as long as wide, with short median ridge anteriorly. Elytra 6.6–6.8 mm long, 5.0–5.2 mm wide; 2.8–2.9 times as long as pronotum; 1.3–1.4 times as wide as pronotum; punctures as large as the pronotal ones, densely distributed; sides strongly curved, widest near 1/2 length of elytron; humeri weakly prominent. Each elytron with two irregular transverse maculae. Anterior elytral macula located behind humerus, in form of arcuate irregular band; anterior margin deeply emarginate; posterior margin weakly emarginate or nearly straight; outer margin not touching lateral margin of elytra, inner margin distant from elytral suture. Posterior macula located at apical 1/3, in form of nearly straight band; outer and inner margin of macula distant from both elytral lateral margin and suture. Both mesotibiae (Fig. 4C) in male with small sharp tooth near apical 1/4 on inner edge, in female without teeth. Pro- and metatibiae simple.

***Abdomen*.** Ventrite 1 longer than 2 and 3 combined; ventrites 2–4 subequal in length. Ventrite 5 (Fig. 5C) arcuate in both sexes. Aedeagus (Fig. 6C) short and stout, heavily sclerotised, curved. Median lobe with two short branches, acute apically. Tegmen basal, ring-shaped.

#### Distribution.

China: Xizang and Yunnan.

### Sinocymbachus wangyinjiei

Taxon classificationAnimaliaColeoptera

Chang & Bi sp. nov.

A932FB08-DD78-5B0B-BE66-50136DBB3F07

http://zoobank.org/1040CEF1-2A3C-4FD2-A9E5-FF27D3490BA3

Figures 1D , 2D , 3D , 4D , 5D , 6D

#### Type material.

***Holotype*.** Male, China: Hubei Province: Shennongjia, 1635 m, 4.X.2007, Yin-Jie Wang leg. (SHNU).

#### Etymology.

The name is dedicated to Mr. Yin-Jie Wang, who collected the holotype of this species for our study.

#### Diagnosis.

*Sinocymbachus wangyinjiei* sp. nov. resembles *S. angustefasciatus*, *S. fanjingshanensis* sp. nov., *S. longipennis* sp. nov., and *S. quadriundulatus*. However, it can be differentiated from *S. angustefasciatus* by the body shiny (vs. opaque); mesotibia in male with deeply U-shaped excision (vs. not excised); from *S. fanjingshanensis* sp. nov. by the anterior margin of mesoventral process much wider than posterior margin (vs. anterior margin as wide as posterior margin); mesotibia in male with deep and narrow excision (vs. with shallow and wide excision); from *S. longipennis* sp. nov. by the body dark brown and shiny (vs. black, opaque); elytra oval with sides distinctly curved (vs. long oval, nearly parallel); mesotibia in male with deep and narrow excision (vs. with shallow and wide excision); from *S. quadriundulatus* by the body without cupreous sheen (vs. with cupreous sheen); mesotibia in male with deeply U-shaped excision (vs. not excised).

#### Description.

Length 9.7 mm. Body oval, approximately 2.1 times as long as wide; convex; shiny. Colour black, shiny, with four orange irregular transverse maculae on elytra.

***Head*.** Antenna (Fig. 2D) long, rather slender, extending to approximately 1/2 body length, with antennomeres 1–8 distinctly longer than wide; scape 5.5 times as long as pedicel; pedicel wider than long; antennomere 3 nearly as long as 4 and 5 combined; antennomeres 4–8 gradually shorter; club moderately broad and flat, loose; antennomeres 10 and 11 with wide concavity ventrally.

***Thorax*.** Pronotum 2.1 mm long, 3.2 mm wide; widest at base; rather coarsely and densely punctate; lateral and anterior margins narrowly bordered; anterior edge with large stridulatory membrane; sides undulate, deeply emarginate near basal 1/4 length; anterior angles bluntly produced; posterior angles acutely produced; disc weakly convex; median furrow absent; lateral sulci short and deep, in form of triangular dent; basal sulcus sinuate, moderately deep. Prosternal process (Fig. 3D) moderately widely separating procoxae; sides curved outwardly toward apex, forked apically, not extending beyond front coxae. Mesoventral process (Fig. 3D) nearly pentagonal, distinctly longer than wide; sides converging to apex; disc with short median ridge anteriorly. Elytra 7.2 mm long, 4.6 mm wide; 3.4 times as long as pronotum; 1.4 times as wide as pronotum; punctation coarse and dense; sides curved, widest near 1/2 length of elytron; converging from here to apex; humeri weakly prominent. Each elytron with two irregular transverse maculae: anterior elytral macula located on humerus, nearly W-shaped; outer margin touching lateral margin of elytra, inner margin distant from elytral suture; posterior macula located at apical 1/3, transverse bands with four distinct projections; outer margin distant from lateral margin of elytra, inner margin distant from elytral suture. Left mesotibia (Fig. 4D–1) in male with small sharp tooth behind 1/2 length on inner edge, then deeply U-shaped excised; right mesotibia (Fig. 4D–2) in male with small sharp tooth near apical 1/3 on inner edge, then deeply U-shaped excised. Pro- and metatibiae simple.

***Abdomen.*** Ventrite 1 almost as long as two subsequent ventrites combined; ventrites 2–4 subequal in length. Ventrite 5 (Fig. 5D) with posterior margin gently rounded. Aedeagus (Fig. 6D) short and stout, heavily sclerotised, straight. Median lobe very short, with two wide, large and rather flat branches. Tegmen basal, ring-shaped.

#### Distribution.

China: Hubei.

### Sinocymbachus angustefasciatus

Taxon classificationAnimaliaColeoptera

(Pic, 1940)

1366539C-8DBE-5A54-8EAA-5FB514D8F21B

Figures 12A , 13A , 14A , 15A , 16A , 17A , 18A

Engonius angustefasciatus Pic, 1940: 11.
Sinocymbachus angustefasciatus : Strohecker and Chûjô 1970: 517.

#### Diagnosis.

*Sinocymbachus angustefasciatus* is most similar to *S. fanjingshanensis* sp. nov., *S. longipennis* sp. nov., *S. quadriundulatus* and *S. wangyinjiei* sp. nov. by having transverse elytral maculae. However, *S. angustefasciatus* differs from *S. fanjingshanensis* sp. nov. by the body opaque (vs. body shiny); left mesotibia in male not excised (vs. widely excised); from *S. longipennis* sp. nov. by the oval elytra with sides distinctly curved (vs. long oval, nearly parallel); left mesotibia in male not excised (vs. widely excised); from *S. quadriundulatus* by the body opaque (vs. body shiny); mesotibial tooth in male symmetric (vs. asymmetric); from *S. wangyinjiei* sp. nov. in having the body opaque (vs. body shiny); mesotibia in male not excised (vs. deeply excised).

#### Length.

9.2–10.6 mm; width: 4.9 mm.

#### Material examined.

**China: Sichuan Province.** Qingchuan County, 13.VII.2013, Jun-Xia Zhang leg. (1 female, MHBU); Wolong Nature Reserve, 18.VII.2013, Yun-Xia Zhang leg. (1 female, MHBU); Wolong, 6–8.VIII.2004, Xiu-Juan Yang & Hui-Ran Hua leg. (2 males, MHBU); Kangding, Pengta, 28.VIII.2005, Fu-Ming Shi leg. (10 males, MHBU); ditto except 29.VIII.2005 (17 males, MHBU); ditto except 30.VIII.2005 (5 males, MHBU); ditto except 31.VIII.2005 (7 males MHBU); ditto except 1.IX.2005 (24 males, MHBU); Yajiang, Decha, 7.IX.2005, Fu-Ming Shi leg. (2 males, MHBU); Jiulong, Hongba, 23.IX.2008, Fu-Ming Shi leg. (1 male, MHBU); ditto except 25.IX.2008 (1 female, MHBU); Kangding, alt. 2624 m, 31.VII.2010, Fu-Ming Shi & Yong-Sheng Pan leg. (1 male, MHBU); Shimian Conty, Liziping, 13.VIII.2010, Fu-Ming Shi leg. (2 males, MHBU); Baoxing Country, Ganyanggou, 30°24'N, 102°38'E, alt. 2000 m, 28.VI.2012, Huang Hao leg. (1 female, SHNU); Shimian Country, Liziping, 28°55'N, 102°13'E, alt. 2600 m, 15.VII.2012, Peng, Dai & Yin leg. (1 female, SHNU)；Shimian Country, Caoke Township, Tuanjie Village, 25.VIII.2016, Jian-Yue Qiu & Hao Xu leg. (1 male, CCLX); Fengtongzhai, Mahuanggou, 30.VII.2016, Cai-Xia Yuan leg. (1 female, MHBU); ditto except 31.VII.2016 (2 males, 2 females, MHBU); Liziping, Zimaping, 31.VII.2016, Cai-Xia Yuan et al. leg. (1 female, MHBU); Kangding, 2500–2700 m, 18.VIII.2014, Wen-Xuan Bi leg. (6 males, 9 females, CBWX); Tianquan County, Labahe, 2060 m, 28–30.VII.2007, Liu, Zhang, Zhou & Bi leg. (2 males, 1 female, CBWX); Emeishan, Huayanding, 1914 m, 15.VIII.2011, Hao Huang leg. (1 male, CCCC); Baoxing, Jiajinshan, Mahuanggou, 27.VI.2012, Xiao-Dong Yang leg. (1 male, CCCC); **Yunnan Province**: Yiliang, Xiaocaoba, 24.VIII.2013, Xun Bian & Guang-Lin Xie leg. (1 male, MHBU); Chongzhou, Jiguanshan, Shaoyaogou, 29.V.2016, Fu-Ming Shi leg. (3 males, 1 female, MHBU); ditto except 31.V.2016 (5 males, MHBU); Shimian, Liziping Protection Station, Gongyihai Station, Liu et al. leg. (1 male, SHEM 24348709); Tianquan County, Labahe, alt. 2100 m, 28–30.VII.2007, Liu et al. leg. (1 female, SHEM24348710); ditto except (1 male, SHEM24348711); ditto except (1 male, SHEM24348712); Shimian Hsien, Liziping N.R., Gongyihai Refuge, 29°01'30.76"N, 102°23'05.11"E, 2056 m, mixed leaf litter, sifted, 25.VII.2016, Zhou, Jiang, Liu & Gao leg. (3 males, 4 females, SHNU); Shimian Hsien, Liziping N.R., Gongyihai-Mamadi, 28°59'24.55"N, 102°24'33.92"E, 2056–2615m, 24.VII.2016, Zhou, Jiang, Liu & Gao leg. (3 males, 2 females, SHNU); Baoxing Hsien, Fengtongzhai N.R., Dashuigou, 30°34'21.95"N, 102°52'54.92"E, 1594 m, 31.VII.2016, Zhou, Jiang, Liu & Gao leg. (1 male, SHNU); **Shaanxi Province**: Ningshan, Pingheliang, 33.479148N, 108.491827E, alt. 2388m, 15.VIII.2013, Xi-Chao Zhu & Ying Tian leg. (2 males, MHBU); Liuba, Caishenmiao, 33°43'27.0"N, 107°12'11.1"E, alt. 1212m, 17.VIII.2013, Xi-Chao Zhu & Ying Tian leg. (1 female, MHBU).

#### Distribution.

China: Sichuan. First records from Yunnan and Shaanxi Provinces of China.

### Sinocymbachus bimaculatus

Taxon classificationAnimaliaColeoptera

(Pic, 1927)

618AF724-574F-51E9-AE31-CD17E2272E03

Figures 7A , 8A, C, E , 12B , 13B , 14B , 15B , 16B , 17B , 18B

Amphisternus bimaculatus Pic, 1927: 11.
Cymbachus bimaculatus : Strohecker 1953: 90.
Sinocymbachus bimaculatus : Strohecker and Chûjô 1970: 513.

#### Diagnosis.

**Figure 7.**
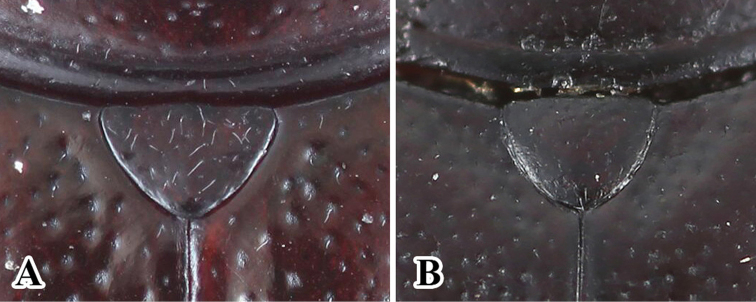
Scutellum. **A***Sinocymbachus bimaculatus***B***Sinocymbachus humerosus*.

*Sinocymbachus bimaculatus* is most similar to *S. humerosus* in appearance, sharing two round maculae on each elytron. However, *S. bimaculatus* differs from *S. humerosus* by the scutellum (Fig. 7) being distinctly longer than wide (vs. nearly as long as wide); and mesotibial tooth in male placed near apical 1/3 length of tibia (vs. near 1/4 length).

#### Length.

8.0–9.7 mm; width: 5.1–5.8 mm.

#### Material examined.

**China: Guizhou Province**: Xishui, Dabaitang, 25–29.IX.2000, Guo-Dong Ren leg. (1 female, MHBU); ditto except 30.IX.2000 (1 female, MHBU); Yanhe County, Mayanghe, Maojia Village, 5–12.VI.2007, Feng-Yan Wang leg. (2 females, MHBU); Fenghuangshan Mt., 27°42'N, 106°55'E, alt. 900 m, 6.III.2012, LI Runyu leg. (1 female, SHNU); Libo Hsien, Maolan N.R., 25°16'38"N, 107°55'29"E, mixed leaf litter, sifted, 800 m, 19.VII.2015, Chen, He, Hu, Wang & Zhao leg. (1 male, 1 female, SHNU); **Guangxi Province**: Shangsi County, Hongqi, 29.XI.2001, Xiu-Juan Yang & Ai-Min Shi leg. (1 female, MHBU); Longsheng County, Huaping, 15.X.2005, Ji-Liang Wang & Chao Gao leg. (1 female, MHBU); Huanjiang County, Yangmeiao Protection Station, 15.VIII.2016, Ling-Xiao Chang leg. (4 males, 1 female, MHBU); Lin’gui Country, Huaping N.R., Anjiangping, alt. 1200 m, 13.VII.2011, He W.-J. & Tang L. leg. (1 female, SHNU); Hechi City, Mulun N.R., 25°8'54"N, 108°2'37"E, mixed leaf litter, sifted, 350–450m, 24.VII.2015, Chen et al. leg. (1 male, 1 female, SHNU); Huanjiang, Jiuwanshan, Yangmeiao, 1200 m, 18.VII.2015, light, Liu & Zhu leg. (1 male, SHEM24344878); Jinxiu, Changtanhe, 860 m, 15.VII.2014, Xiao-Bin Song leg. (1 male, CBWX); Maoershan, Lijiangyuan, 810 m, 28.VII.2014, Xiao-Bin Song leg. (2 females, CBWX); Jinxiu, Houzishan, 960 m, 13.VII.2014, Xiao-Bin Song leg. (1 male, CBWX); **Sichuan Province**: Luzhou, Huangjing, 20.VII.2002, Ming Bai & Jian-Feng Wang leg. (1 female, MHBU); **Hunan Province**: Zhangjiajie, Sangzhi, Guandiping, day, 16.VII.2010, Hao Xu leg. (1 male, MHBU); ditto except 28.VII.2010 (6 females, MHBU); Anhua County, Liubu, 16–17.VII.2004, Ji-Liang Wang leg. (1 male, MHBU); Tongdao County, Mujiao, 25.VII.2004, Jiang-Feng Wang & Ji-Liang Wang leg. (1 male, 1 female MHBU); Yongshun County, Xiaoxi, 8.VIII.2004, Ji-Liang Wang leg. (1 male, 1 female, MHBU); Yueyang City, Pingjiang County, Fushoushan, 28°28'N, 113°46'E. 1079 m, 18-26.VII.2016, Jiang-Jiang Liu & Zhou leg. (1 female, SHNU); Dongan County, Damiaokou Town, Shunhuangshan Park, Ehuangxi, 8.X.2015, Chi Jin leg. (CCLX); **Zhejiang Province**: Quzhou City, Jiangshan County, Shuangxikou, 27°55'02.72"N, 119°11'34.47"E, alt. 496 m, mixed leaf litter, sifted, 12.VIII.2018, Cheng & Miao leg. (1 female, SHNU); Kaihua, Gutianshan, 500–850 m, 21.IV.2013, Xiao-Bin Song leg. (5 males, 5 females, CBWX); **Fujian Province**: Wuyishan, Taohuayu, 9.VI.2013, Chi Jin & Jie Yang leg. (1 male, MHBU); Jianning, Jinraoshan, 12.VI.1956, Gen-Tao Jin & Yang-Ming Lin leg. (1 female, SHEM24295200); ditto except (1 male, SHEM24295201); ditto except (1 female, SHEM24295202); ditto except (1 male, SHEM24295203); ditto except (1 female, SHEM24295204); ditto except (1 male, SHEM24295205); ditto except (1 female, SHEM24295207); ditto except (1 male, SHEM24295208); ditto except (1 female, SHEM24295209); ditto except (1 male, SHEM24295210); ditto except (1 female, SHEM24295211); ditto except (1 female, SHEM24295212); ditto except (1 female, SHEM24295213); ditto except (1 female, SHEM24295214); ditto except (1 female, SHEM24295216); ditto except (1 female, SHEM24295223); ditto except (1 male, SHEM24295226); ditto except (1 female, SHEM24295228); ditto except (1 female, SHEM24295229); ditto except (1 female, SHEM24295199); Nanping City, Mangdangshan Mt., 26°41'51"N, 118°07'00"E, mixed forest, leaf litter, sifted, 400 m, 10.XI.2015, Yan & Tang leg. (1 female, SHNU); **Guangdong Prvince**: Nanling, 8.V.2008, Hong-Liang Shi leg. (2 males, 1 female, MHBU); Lianxian, Dadongshan, 28.V.1997 Chen Hong leg. (1 female, SYSU En-131910); Lianxian, Dadongshan, 3.IX.1994 Chang-Ping Zhao leg. (1 female, SYSU En-131911); Lianzhou City, Dadongshan, 2.VI.1998 Han Zhang leg. (1 female, SYSU En-131912); Lianxian, Dadongshan, 27.V.1997 Xiao-Xin Zhang leg. (1 female, SYSU En-131913); ditto except Ji Zheng leg. (1 female, SYSU En-131915); ditto except, 28.V.1997 Jian-Hua Li leg. (1 male, SYSU En-131916); Fengkai, Heishiding, 3.VII.1986, Zhen-Ao Chen leg. (1 female, SYSU En-131928); **Hainan Province**: Ledong Country, Jianfengling, Mingfenggu, 18°44'N, 108°50'E, alt. 950m, 30.IV.2012, PENG & DAI leg. (1 female, SHNU); Qiongzhong Country, Limushan, 1160 m, 4.IV.2016, Ling-Xiao Chang & Xing-Long Bai leg. (1 female, CCLX).

#### Distribution.

Vietnam: Tonkin. China (new country record): Guizhou, Guangxi, Sichuan, Hunan, Zhejiang, Fujian, Guangdong and Hainan.

#### Biology and ecology.

**Figure 8.**
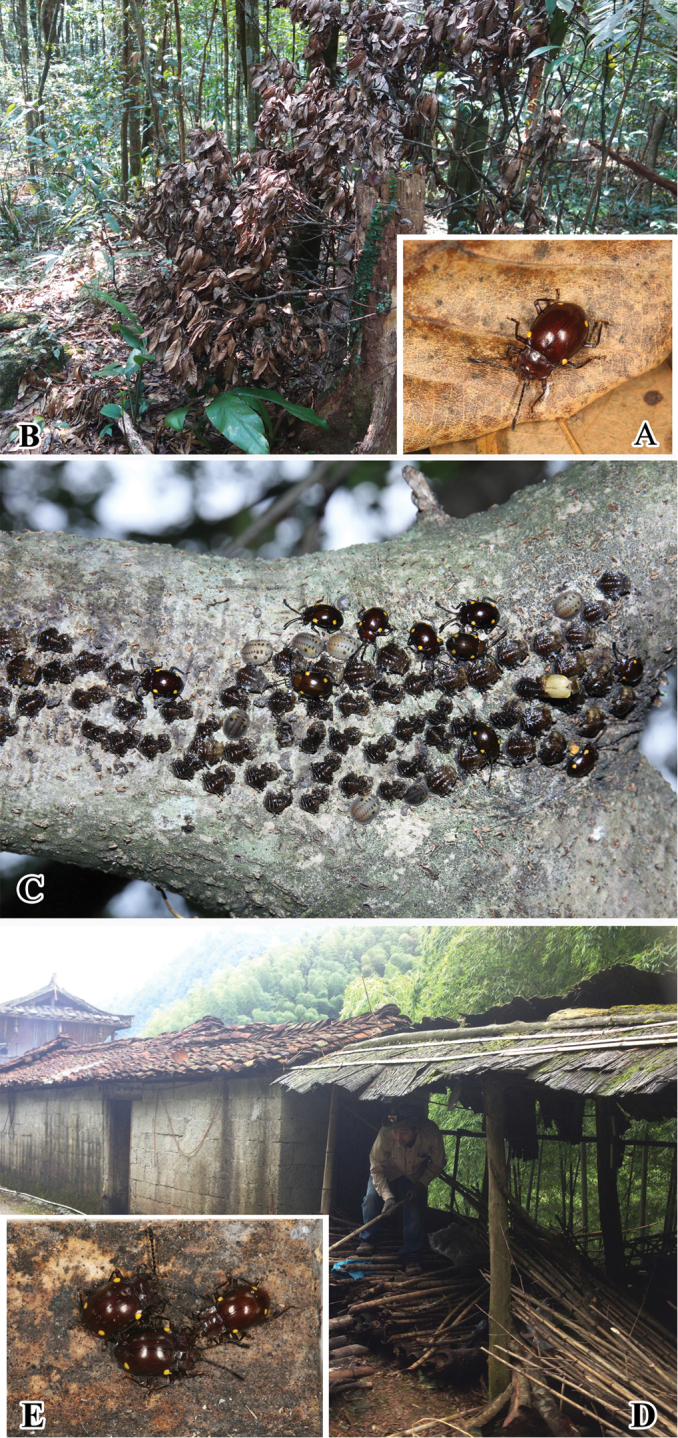
Habitats of *Sinocymbachus bimaculatus***A, E** adult of *S. bimaculatus***B** large clump of Fagaceae plants at collection site in Hainan, China **C** clusters of mature larvae, prepupae or pupae of *S. bimaculatus*, with newly emerged adults, beneath the tree trunk **D** search for Endomychidae from large pile of dead bamboos in Guangxi, China.

The adults were collected by shaking the tree from a large clump of dead wood of Fagaceae plants (Mt. Limushan, Qiongzhong, Hainan) (Fig. 8A, B). The adults and larvae were hand collected from a large pile of dead bamboos (Yangmeiao, Huanjiang, Guangxi) (Fig. 8D, E). The adults and larvae apparently feed together (Fig. 8C). *Sinocymbachus bimaculatus* is not only most similar to *S. humerosus*, but both are also sympatric.

### Sinocymbachus decorus

Taxon classificationAnimaliaColeoptera

Strohecker & Chûjô, 1970

E9D2F9E2-A691-5274-90B5-E239143AFD8D

Figures 12C , 13C , 14C , 15C , 16C , 17C , 18C

Sinocymbachus decorus Strohecker & Chûjô, 1970: 516.

#### Diagnosis.

*Sinocymbachus decorus* is most similar to *S. luteomaculatus* and *S. politus* in appearance: elytra in both species have two basal spots and one narrow transverse apical band. However, *S. decorus* differs from *S. luteomaculatus* by having a shiny body (vs. body opaque); humeri roundly subcarinate, prominent (vs. weakly prominent); from *S. politus* by the body without cupreous sheen (vs. with cupreous sheen); two basal elytral maculae arranged in oblique line (vs. spots arranged in horizontal line); mesotibial tooth in male asymmetric (vs. symmetric).

#### Length.

10.4–10.6 mm; width: 5.1–5.5 mm.

#### Material examined.

**China: Yunnan Province**: Lincang, Wulaoshan Forest Farm, 23°54'36.4"N, 100°11'04.3"E, alt. 2371 m, 8–10.VII.2009, Ji-Shan Xu & Li-Xiang Zhang leg. (2 females, MHBU); Lincang, Wulaoshan Forest Farm, Qingrengu, 25.VIII.2019 D, Ling-Xiao Chang leg. (1 male, 1 female, CCLX); Yun County, Yongbao Town, Pinghe Reservoir, X.2018, Zi-Chun Xiong leg. (1 male, 1 female, CCLX); Jingdong County, Ailaoshan, 7–9.VIII.2009, Ji-Shan Xu & Zhong-Kun Li leg. (1 male, MHBU); Lushui, Yaojiaping, 2700 m, 21.VI.2010, Wen-Xuan Bi leg. (2 males, CBWX); ditto except 2450 m, 4.V.2015 (2 males, 1 female, CBWX); ditto except 13.VIII.2015 (1 male, 1 female, CBWX); ditto except 2450–2700 m, 14.IV.2018 (1 male, 1 female, CBWX); ditto except 2500 m, 15.IX.2018 (1 male, CBWX); Lushui, Pianma, Gangfang, 2100 m, 7.VI.2015, Wen-Xuan Bi leg. (1 male, CBWX); Dayao County, Santai Town, Xiaobaicaoling, 2980 m, 29–30.V.2013, Wen-Xuan Bi leg. (1 female, CBWX); Nanjian Sheyaojing, 2450 m, 11.VII.2017 em VII.21, Wen-Xuan Bi leg. (1 female, CBWX); Tengchong, Houqiao, Heinitang, 11–14.IX.2018, Wen-Xuan Bi leg. (2 females, CBWX); Gongshan, Shaunglawa, 1650 m, 17.VI.2015, Wen-Xuan Bi leg. (1 female, CBWX).

#### Distribution.

China: Yunnan.

#### Biology and ecology.

The adults were collected from dead leaves of Fagaceae plants by shaking the tree (Mt. Wulaoshan, Lincang, Yunnan).

### Sinocymbachus excisipes

Taxon classificationAnimaliaColeoptera

(Strohecker, 1943)

2BEF80D0-E678-50E5-8E70-5DF626A034BF

Figures 9 , 12D , 13D , 14D , 15D , 16D , 17D , 18D

Engonius excisipes Strohecker, 1943: 383.
Cymbachus excisipes : Strohecker 1953: 90.
Sinocymbachus excisipes : Strohecker and Chûjô 1970: 515.

#### Diagnosis.

**Figure 9.**
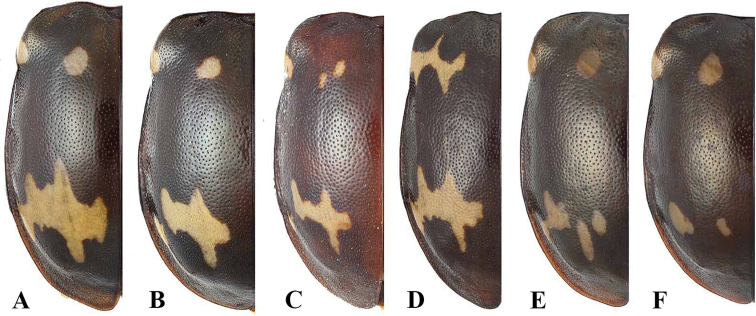
Variation in elytral maculae of *Sinocymbachus excisipes*. **A** general morph **B–F** variants morphs.

*Sinocymbachus excisipes* can be separated from all its congeners by having the intercoxal process of mesoventrite with large tubercle instead of short medina ridge at base; ventrite 5 with posterior margin abruptly projecting medially in male. Furthermore, the basal and apical elytral maculae varied in different specimens - they may be composed of 2–4 small spots, or form transverse bands with strong dentations/projections (Fig. 9).

#### Length.

9.5–10.9 mm; width：6.0–6.2 mm.

#### Material examined.

**China: Zhejiang Province.** Tianmushan, 370–1100 m, 5.VIII.1962, Gen-Tao Jin leg. (1 female, SHEM 24343460); Longquan, Fengyangshan, 1926 m, 29.VI.2015, Liu et al. leg. (1 male, SHEM 24345681); Tianmushan, 11–14.VIII.1987, Wu Wu leg. (1 female, SYSU En-096206); Linan, Xitianmushan, 1100 m, 1–9.VII.2006, Wen-Xuan Bi leg. (1 male, 1 female, CBWX); ditto except 29.VI–1.VII.2007 (1 male, CBWX); ditto except 1100–1050 m, 14.VII.2016 (3 males, 3 females, CBWX); ditto except 10-16.VII.2018 (48 males, 24 females, CBWX); **Hunan Province**: Sangzhi County, near Tianpingshan Control Station, 15.IX.2015, Chi Jin leg. (1 male, CCLX); Yichang City, Wufeng Hsien, Houhe N. R., 3.VIII.2013, Hao Huang leg. (1 female, SHNU); **Chongqing Province**: Qianjiang District, Shuishicun, 8.VII.2011, Qiang Guo leg. (1 male, MHBU); **Guizhou Province**: Daozhen, Dashahe, 24.VIII.2004, Fu-Ming Shi leg. (1 female, MHBU); **Sichuan Province**: Emeishan, alt. 890 m, 28.VI.2009, Yu-Ting Chen leg. (2 females, MHBU); Emeishan, Leiyinsi, 11.VIII.2011, Fu-Ming Shi & Le-Hong Zhao leg. (1 male, MHBU); Eemeishan, Xixinsuo, 1400 m, 28.VI.2018, Wen-Xuan Bi leg. (1 female, CBWX); Wolong, 6–8.VIII.2004, Xiu-Juan Yang & Hui-Ran Hua leg. (3 males, 3 females, MHBU); Dujiangyan, Qingchengshan, 11–12.VIII.2004, Xiu-Juan Yang & Hui-Ran Hua leg. (1 female, MHBU); **Guangxi Province**: Jinxiu, Dayaoshan, Pingbanshan, 1150 m, 18.VII.2016, Jin-Teng Zhao leg. (2 females, CBWX); **Fujian Province**: Wuyishan, 541 m, VI.2018, leg. Zhu-Qing He (1 male, CBWX).

#### Distribution.

China: Sichuan, Hubei. First records from Zhejiang, Hunan, Chongqing, Guizhou, and Guangxi Provinces of China.

### Sinocymbachus humerosus

Taxon classificationAnimaliaColeoptera

(Mader, 1938)

8B5201F3-F932-5913-919E-302250C315D7

Figures 7B , 10 , 12E , 13E , 14E , 15E , 16E , 17E , 18E

Cymbachus humerosus Mader, 1938: 40.
Amphisternus quadrinotatus Chûjô, 1938: 396.
Sinocymbachus humerosus : Strohecker and Chûjô 1970: 512.

#### Diagnosis.

*Sinocymbachus humerosus* is most similar to *S. bimaculatus* in sharing two round maculae on each elytron. However, *S. humerosus* differs from *S. bimaculatus* by having the scutellum (Fig. 7) nearly as long as wide (vs. distinctly longer than wide); and mesotibial tooth in male placed near apical 1/4 of tibial length (vs. near 1/3).

#### Length.

8.4–8.9 mm; width: 4.7–5.3 mm.

#### Material examined.

**China: Zhejiang Province**: Linan, Dajingwu, 9.VI.2012, Ling-Xiao Chang leg. (1 male, MHBU); Suichang County, Jiulongshan Reserve, Yanping, alt. 700–800 m, 12.X.2008, Jun-Hao Huang leg. (3 males, 3 females, MHBU); Longquan, Fengyangshan, 19.VII.2012, Guang-Lin Xie & Xin Wang leg. (1 male, 4 females MHBU); Tianmushan, Dahenglu, 14.VII.2012, Guang-Lin Xie leg. (1 female, MHBU); Longquan, Fengyangshan, 25.VII–1.VIII.2007, Hao-Yu LIU & Zhen-Hua Gao leg. (1 male, MHBU); Lin’an City, Mt. East Tianmushan, alt. 1050–1150 m, 13.IV.2011, Peng & Zhu leg. (1 female, SHNU); Longquan, Fengyangshan, 1250 m, 17.V.2007, Bao-Feng Zhou & Lei Wang leg. (1 female, SHEM24295628); Xitianmushan, 300–600 m, 2.VI.2016, Wen-Xuan Bi leg. (1 female, CBWX); ditto except 450 m, 4.VII.2016 (1 male, CBWX); ditto except 1300 m, 26.VII.2016 (10 males, 9 females, CBWX); ditto except 350 m, 11.VIII.2016, (1 female, CBWX); ditto except 450–350 m, 17.VI.2014 (5 males, 6 females, CBWX); ditto except 350 m, 23.VI.2014 (2 females, CBWX); Tianmushan, 1100 m, 2.V.2005, Wen-Xuan Bi leg. (1 female, CBWX); Anji County, Longwangshan, Shenxi, 250–550 m, 22–26.IV.2006, Wen-Xuan Bi leg. (1 female, CBWX); ditto except 350 m, 7–11.VI.2012 (1 male, CBWX); Longquan, Fengyangshan, Fengyanghu, 1560 m, 5.X.2013, Wen-Xuan Bi leg. (1 female, CBWX); Changhua, Qingliangfeng, 950 m, 13–17.VI.2014, Wen-Xuan Bi leg. (1 female, CBWX); Kaihua, Gutianshan, 500–850 m, 21.VI.2013, Wen-Xuan Bi leg. (7 males, 7 females, CBWX); **Jiangxi Province**: Longnan, Jiulianshan, 23.VII.2008, Fu-Ming Shi & Ming Qiu (1 female, MHBU); Ji’an City, Jinggangshan, Longtan, 26°35'47"N, 114°08'25"E, mixed forest, shrub, flower sweeping & beating, 760–920 m, 29.VII.2014, Chen, Hu, Lv & Yu leg. (1 female, SHNU); **Guangxi Province**: Longsheng, Huaping, 6.VI.1963, Si-Kong Liu leg. (1 female, IZCAS); Lingui, Huaping, 500 m, 30.V.2010, Zheng Li leg. (1 male, 3 females, CBWX); Damingshan, 27.V.2011, Qing Zhang & Hai-Ling Wang leg. (1 male, 1 female, MHBU); Jinxiu, 6K, 24°9'19"N, 110°12'22"E, alt. 1155 m, 8.III.2016, Yu-Yang Lei leg. (4 males, 2 females, CCLX); Fangchenggang, Shiwandashan Reserve, 29.VI.2015, Zhi-Lin Chen leg. (5 males, 2 females, MHBU); Rongshui, Jiuwanshan, 24.VII.2015 N, Ling-Xiao Chang leg. (1 male, 1 female, CCLX); Jinxiu, 1155 m, Yu-Yang Lei leg. (4 males, 2 females, CCLX); Jinxiu, Changtanhe, 860 m, 15.VII.2015, Xiao-Bin Song leg. (1 male, 1 female, CBWX); Jinxiu, Houzishan, 960 m, 13.VII.2014, Wen-Xuan Bi leg. (1 male, CBWX); Maoershan, Manjiangyuan, 810 m, 28.VII.2014, Xiao-Bin Song leg. (1 female, CBWX); Shanglin, Xiyan Town, 29.IV.2017, Yan-Quan Lu leg. (1 male, 3 females, CCCC); **Yunnan Province**: Bannan, Menglun, X.2014, Xiao-Yu Zhu leg. (1 female, MHBU); **Hunan Province**: Tongdao County, Mujiao Township, 25.VII.2004, Jian-Feng Wang & Ji-Liang Wang leg. (5 males, MHBU); Dongan County, Shunhuangshan, 3.X.2004, Jian-Hua Huang leg. (1 male, MHBU); Dongan County, Damiaokou Town, Shunhuangshan Park, 9.X.2015, Chi Jin leg. (2 females CCLX); Suining County, Huangsangping Town, Hongjun Road, 22.IX.2015 Chi Jin leg. (1 female, CCLX); Dao County, Qingtang Township, Laozhongping Village, Yueyan Forest Farm, 30.IX.2015, Chi Jin leg. (1 female, MHBU); **Guizhou Province**: Jiangkou, Heiwan, 3.VIII.2011, Guo-Dong Ren leg. (1 male, MHBU); Libo, Maolan, 21.VII.2015, Chi Jin leg. (1 female, CCLX); **Guangdong Province**: Nanling, 8.V.2008, Xiao-Yu Zhu leg. (1 female, MHBU); Nanling, V-VIII.2008, Hong-Liang Shi leg. (2 females, MHBU); Nanling, 18.VIII.2010, Hao-Yu Liu leg. (1 female, MHBU); Fengkai, Heishiding, 12.X.1984, Zhen-Ao Chen leg. (1 female, SYSU En-131937); ditto except 9.X.1984, Zhi-Hong Zhou leg. (1 male, SYSU En-131939); ditto except 17.X.1984, Han-Chuan Hu leg. (1 male, SYSU En-131940); **Hainan Province**: Wanning, Diaoluoshan, 17–18.XI.2006, Guo-Dong Ren leg. (2 males, 2 females, MHBU); Jiangfengling, alt. 950 m, 15.IV.2010, Xiao-Yu Zhu leg. (1 female, MHBU); Yinggeling, Hongxin Village, 11–13.V.2011, Xiao-Qing Yang & Lin-Fei Wang leg. (2 females, MHBU); Ledong, Jianfengling, 16.XI.2006, Li-Zhen Li leg. (1 female, SHNU); Bawangling, 15.XI.1964, Zhen-Yao Chen leg. (1 female, SYSU); Baisha, Yinggezui Protection Station, 678 m, Guo Zheng leg. (1 female, CCLX); Qiongzhong, Limushan, 1160 m, 4.IV.2016, Ling-Xiao Chang & Xing-Long Bai leg. (2 females, CCLX); Jianfengling, Mingfenggu, 983 m, 29.IV.2014, Chao Wu leg. (1 male, 1 fenale, CBWX); Ledong, Jianfengling, 16.XI.2006, Li-Zhen Li leg. (1 female, SHNU); Jianfengling, Mingfenggu, 950–1000 m, Wen-Xuan Bi leg. (2 males, 2 females, CBWX); **Fujian Province**: Jianning, Jinraoshan, 12.VI.1956, Gen-Tao Jin & Yang-Ming Lin leg. (1 female, SHEM24295206); ditto except (1 female, SHEM24295215); ditto except (1 male, SHEM24295220); ditto except (1 female, SHEM24295221); ditto except (1 female, SHEM24295222); ditto except (1 male, SHEM24295227); Daan, 3.VI.1956, Gen-Tao Jin & Yang-Ming Lin leg. (1 male, SHEM24295230); ditto except (1 female, SHEM24295231); Wuyishan, Taoyuanyu, 6.VIII.2016, Hai-Tian Song leg. (1 male, CCLX); **Taiwan Province**: Taoyuan, Tengzhi, 18.III.1997, Wen-Yi Zhou leg. (1 female, MHBU); Pingdong, Wutai Country, alt. 1450 m, 15.IV.2011, Wen-Yi Zhou leg. (1 female, MHBU); Pingdong, Dahanshan, 25.XII.2007, Chang-Chin Chen leg. (1 male, 1 female, MHBU).

#### Distribution.

China: Jiangsu, Jiangxi, Fujian, Guangdong, Hainan and Taiwan. First records from Zhejing, Hunan, Guangxi, Yunnan, and Guizhou Provinces of China.

#### Remarks.

**Figure 10.**
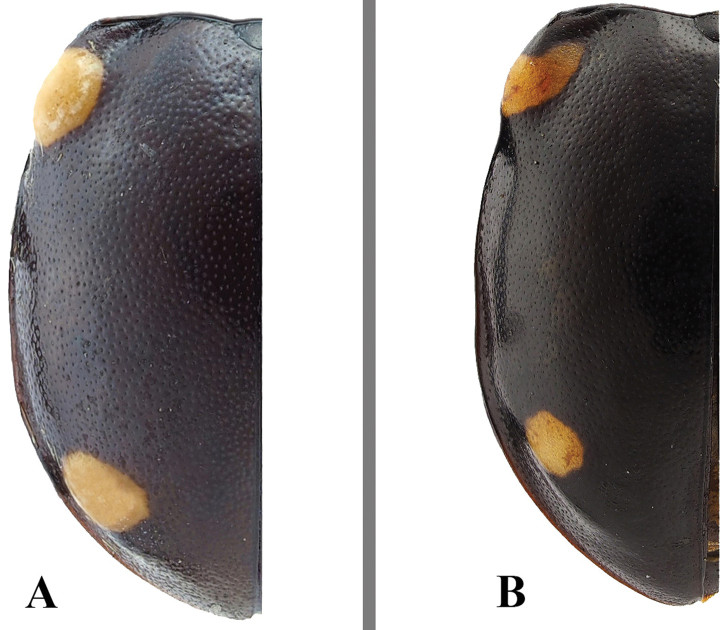
Variation in elytral maculae of *Sinocymbachus humerosus*. **A** specimen from mainland China **B** specimen from Taiwan.

The anterior elytral macula is transverse with anterior margin distinctly emarginate in specimens from Taiwan, China (Fig. 10B).

### Sinocymbachus koreanus

Taxon classificationAnimaliaColeopteraEndomychidae

(Chûjô & Lee, 1993) comb. nov.

6930CEA9-854C-5E81-81D5-65BE51501D74

Cymbachus koreanus Chûjô & Lee, 1993: 95.

#### Diagnosis.

*Sinocymbachus koreanus* can be separated from all its congeners by having the body very short, nearly circular, strongly converging from 1/2 length of elytron to apex; each elytron with four small round spots, two basal spots located posterior to humerus and arranged in a slightly oblique line, and two apical spots located in apical ¼ length, arranged nearly horizontally.

#### Length.

7.5–7.9 mm. Width: 5.1–55 mm.

#### Remarks.

This species was originally described in the geuns *Cymbachus* Gerstaecker, 1857. However, the following characters of this species match the definition of *Sinocymbachus* proposed by Strohecker & Chûjô (1970): mesotibiae sexually dimorphic, toothed on inner edge in male, simple in female (Chûjô and Lee 1993); aedeagus complicated, trilobed (Chûjô and Lee 1993; Boo 2013). Therefore, *Cymbachus koreanus* Chûjô & Lee, 1993 is transferred to *Sinocymbachus*.

#### Distribution.

South Korea.

### Sinocymbachus luteomaculatus

Taxon classificationAnimaliaColeopteraEndomychidae

(Pic, 1921)

6D19F128-4428-5D17-996E-2B5B5CC0FFA2

Figures 12F , 13F , 14F , 15F , 16F , 17F , 18F

Engonius luteomaculatus Pic, 1921: 1.
Cymbachus luteomaculatus : Strohecker 1953: 91.
Sinocymbachus luteomaculatus : Strohecker and Chûjô 1970: 516.

#### Diagnosis.

Based on Strohecker & Chûjô (1970), *Sinocymbachus luteomaculatus* can be separated from all its congeners by having the basal and apical elytral maculae briefly undulately fasciate, but in one specimen examined by the author base of elytron is composed of two spots (Fig. 18F), this elytral pattern is most similar to *S. decorus* and *S. politus* by sharing two basal spots and one narrow transverse apical macula on each elytron. However, *S. luteomaculatus* differs from *S. decorus* by having an opaque body (vs. shiny) and humeri weakly prominent (vs. distinctly prominent); from *S. politus* by the elytra distinctly more elongate (vs. not elongate); two basal elytral maculae arranged in an oblique line (vs. arranged in a horizontal line); mesotibial tooth in male asymmetric (vs. symmetric).

#### Length.

9.6–10.7 mm; width: 5.2–5.5 mm.

#### Material examined.

**China: Yunnan Province**: Zhanyi County, Zhujiangyuan, alt. 2100 m, 18.VII.2010, Ji-Shan Xu & Zhong-Kun Li leg. (1 male, 2 females, MHBU).

#### Distribution.

China: Yunnan.

#### Remarks.

Although few specimens only were available for this work, basal elytral maculae briefly undulately fasciate and the shape of the aedeagus were enough to determine they are the same species (Fig. 17F).

### Sinocymbachus parvimaculatus

Taxon classificationAnimaliaColeopteraEndomychidae

(Mader, 1938)

423FF729-0806-5521-BACE-C7643931EEAA

Figures 12G , 13G , 14G , 15G , 16G , 17G , 18G

Cymbachus parvimaculatus Mader, 1938: 40.
Sinocymbachus parvimaculatus : Strohecker and Chûjô 1970: 512.

#### Diagnosis.

*Sinocymbachus parvimaculatus* can be separated from all its congeners by having the body very short, nearly circular and each elytron possessing two small round spots placed on the mid-line of each elytron.

#### Length.

7.7 mm–8.5 mm; width: 5.6 mm.

#### Material examined.

**China: Yunnan Province: Nanjian, Sheyaojing**, 2150–2300 m, 11.VII.2017, Wen-Xuan Bi & Yu-Tang Wang leg. (1 male, CBWX); Weishan, Weibaoshan, 2200 m 5.VII.2017, Wen-Xuan Bi leg. (1 female, CBWX); Weishan, Weibaoshan, 2300 m, 9.VII.2017, Wen-Xuan Bi leg. (1 female, CBWX); Eshan Country, alt. 1688 m, 28.VII.2009, Ji-Shan Xu & Li-Xiang Zhang leg. (1 female, MHBU); Shizong Country, Junzishan, 16.VII.2006, Jun-Tong Lang & Yu-Xia Yang leg. (1 female, MHBU); Lincang City, 1900 m, IV. 2016, Zi-Chun Xiong leg. (1 female, CCLX).

#### Description of male.

Length 7.8 mm. Body short oval, approximately 1.5 times as long as wide; strongly convex; smooth. Colour black with four orange round spots on elytra.

***Head*.** Antenna composed of 11 antennomeres, rather stout, extending to ca. 1/2 body length, with antennomeres 1–8 distinctly longer than wide; scape 4.0 times as long as pedicel; antennomere 3 as long as antennomeres 4 and 5 combined; antennomere 4 as long as antennomere 5; antennomeres 5–8 gradually shorter; club composed of three antennomeres, wide, approximately 3.0 times as wide as antennomere 8, moderately flat, rather loose.

***Thorax*.** Pronotum 1.5 mm long, 3.5 mm wide; widest at base; moderately coarsely and rather densely punctate; lateral and anterior margins narrowly bordered; anterior edge with moderately large stridulatory membrane; sides weakly undulate; anterior angles produced, bluntly rounded; posterior angles moderately acutely produced; disc weakly convex; median furrow absent; lateral sulci very short and deep, in form of triangular dent; basal sulcus curved, moderately deep. Prosternal process moderately widely separating procoxae; sides curved outwardly near apex, forked apically, not extending beyond front coxae. Mesoventral process nearly pentagonal, as long as wide, with short median ridge near basal 1/4. Elytra 5.9 mm long, 5.3 mm wide; 3.9 times as long as pronotum; 1.5 times as wide as pronotum; punctures as large as the pronotal ones, more dense; sides strongly curved, widest near 1/2 length of elytron; humeri weakly prominent. Each elytron with two round maculae: anterior macula located near basal 1/5, posterior macula located at apical 1/3, and all these maculae placed in mid-line of elytron; outer and inner margins of macula very distant from elytral lateral margin and suture. Mesotibia with small sharp tooth near apical 1/4 on inner edge, then with deep, small, U-shaped excision. Pro- and metatibiae simple.

***Abdomen*.** Ventrite 1 longer than 2 and 3 combined; ventrites 2–4 subequal in length. Ventrite 5 with lateral margins gradually converging posteriorly; posterior margin broadly rounded medially. Aedeagus (Fig. 18) short and stout, heavily sclerotised, curved. Median lobe with two short branches, simple, acute apically. Tegmen basal, ring-shaped.

#### Distribution.

China: Yunnan.

### Sinocymbachus politus

Taxon classificationAnimaliaColeopteraEndomychidae

Strohecker & Chûjô, 1970

6A584423-A627-5079-A14D-51B1ED2E1DF3

Sinocymbachus politus Strohecker & Chûjô, 1970: 515

#### Diagnosis.

Based on Strohecker & Chûjô (1970), *S. politus* is most similar to *S. decorus* and *S. luteomaculatus* in appearance: both species share the elytra with two basal spots and one narrow transverse apical band. However, the body with cupreous sheen, two basal elytral maculae arranged in horizontal line and mesotibial tooth in male symmetric can separate it from all these similar species.

#### Length.

9.3 mm.

#### Distribution.

China: Taiwan.

#### Remarks.

The diagnosis of *S. politus* is based on its original description of Strohecker & Chûjô (1970) due to a lack of material for study.

### Sinocymbachus quadrimaculatus

Taxon classificationAnimaliaColeopteraEndomychidae

(Pic, 1927)

DCE1DF5C-81AB-5710-960E-1FF44566FA17

Figures 11 , 12H , 13H , 14H , 15H , 16H , 17H , 18H

Amphisternus quadrimaculatus Pic, 1927: 11.
Cymbachus quadrimaculatus : Strohecker 1953: 91.
Sinocymbachus quadrimaculatus : Strohecker and Chûjô 1970: 515.

#### Diagnosis.

*Sinocymbachus quadrimaculatus* differs from all its congeners in having each elytron with four small round spots, two basal spots located posterior to humerus and arranged horizontally, and two apical spots located in apical ¼ length, arranged in oblique line.

#### Length.

14.0–16.2 mm; width: 8.4–8.5 mm.

#### Material examined.

**China. Zhejiang Province**: Jiangshan City, Hongyanding, 11.VIII.2016, Yi-Bin Ba & Ling-Xiao Chang leg. (1male, 1 female, MHBU); **Guangxi Province**: Jinxiu, Changtong, Dayaoshan, 23.V.2019, Chun-Fu Feng leg. (4 males, 6 females, CCLX); Xingan, Huanjiang, 5.VII.2006 (1 male, CCLX); Jinxiu, Yinshan Protection Station, 1200 m, 9.VII.2014, Xiao-Bin Song leg. (1 female, CBWX); Laibin, Jinxiu, Dayaoshan, 1017 m, 16.V.2015, Yan-Quan Lu leg. (1 male, 1 female, CCCC); **Hunan Province**: Tongdao County, Mujiao Township, 26.VII.2004, Ji-Liang Wang leg. (2 males, 2 females, MHBU); Sangzhi County, near Tianpingshan Control Station, 15.IX.2015, Chi Jin leg. (1 male, CCLX); **Hainan Province**: Bawangling, 9.VII.2006, Ji-Liang Wang & Chao Gao leg. (1 male, 5 females, MHBU); Wuzhishan, 750 m, 15.X.2014, Chao Wu leg. (1 female, CBWX); **Fujian Province**: Ningde, Nanjiao, Houshancun, 200–300 m, 3–5.X.2012, De-Yao Zhou leg. (1 male, 1 female, CBWX).

#### Distribution.

Vietnam: Tonkin; China: Fujian. First records from Zhejiang, Guangxi, Hunan and Hainan Provinces of China.

#### Remarks.

**Figure 11.**
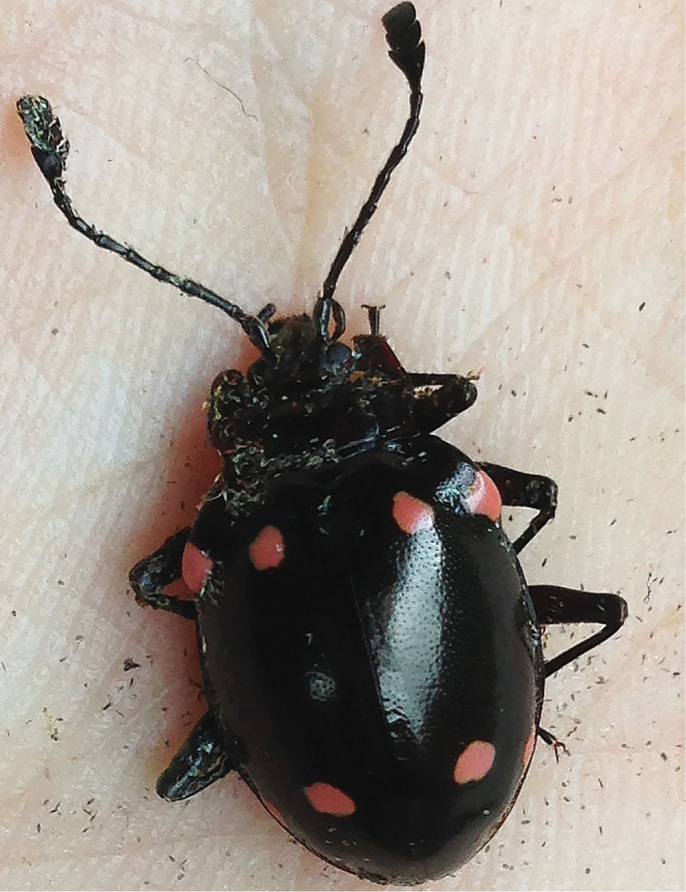
Live adult of *Sinocymbachus quadrimaculatus* from Zhejiang, China.

Some live or fresh specimens from Zhejiang or Fujian were observed with the elytral maculae pink in colouration (Fig. 11), which gradually turned yellow after drying. However, elytral maculae in the specimens collected from Hainan are always yellow.

**Figure 12.**
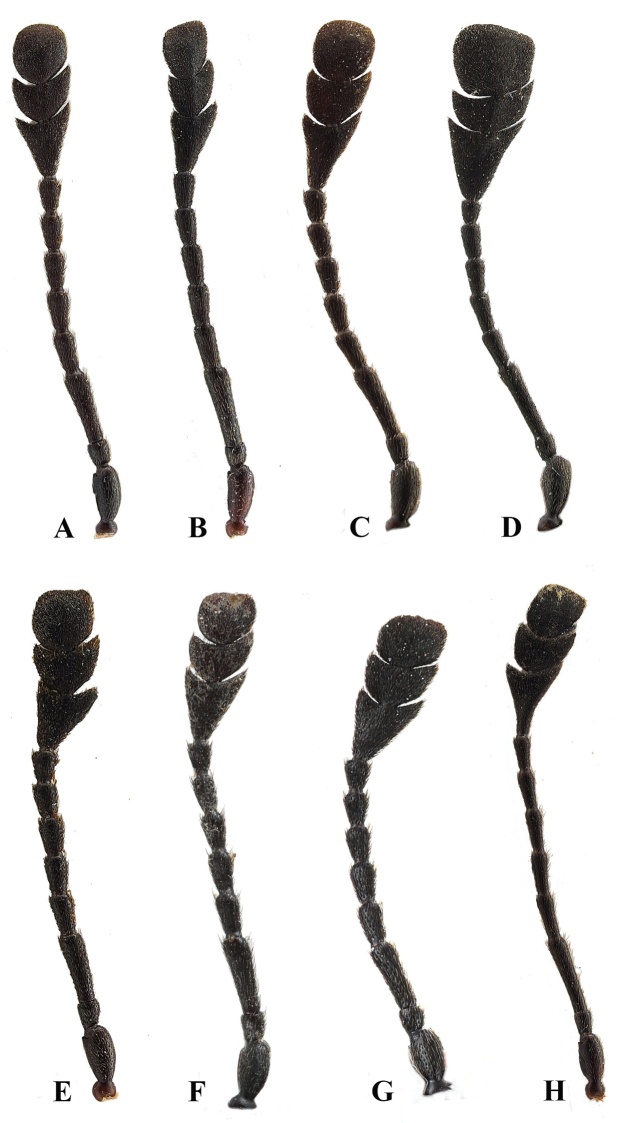
Left antenna of *Sinocymbachus* spp. (dorsal view) **A***S. angustefasciatus***B***S. bimaculatus***C***S. decorus***D***S. excisipes***E***S. humerosus***F***S. luteomaculatus***G***S. parvimaculatus***H***S. quadrimaculatus*.

**Figure 13.**
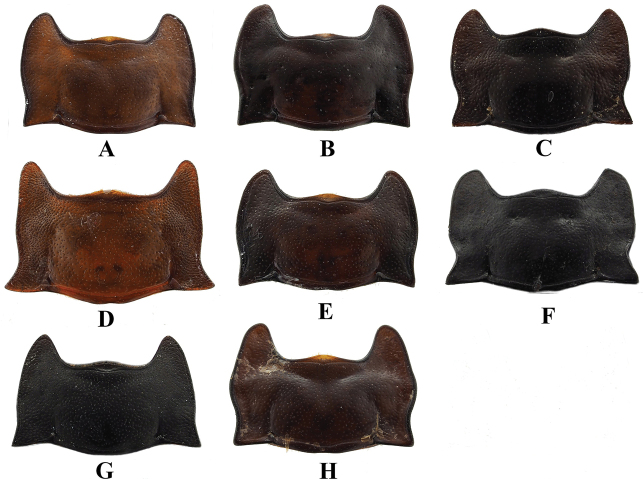
Pronotum of *Sinocymbachus* spp. **A***S. angustefasciatus***B***S. bimaculatus***C***S. decorus***D***S. excisipes***E***S. humerosus***F***S. luteomaculatus***G***S. parvimaculatus***H***S. quadrimaculatus*.

**Figure 14.**
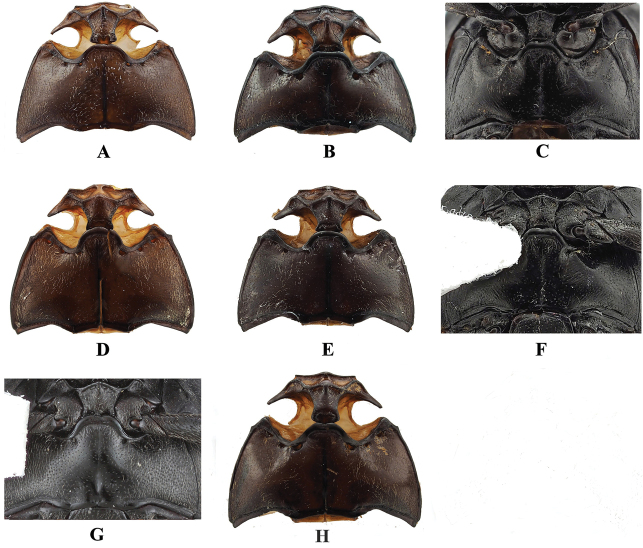
Meso- and metaventrite of *Sinocymbachus* spp **A***S. angustefasciatus***B***S. bimaculatus***C***S. decorus***D***S. excisipes***E***S. humerosus***F***S. luteomaculatus***G***S. parvimaculatus***H***S. quadrimaculatus*.

**Figure 15.**
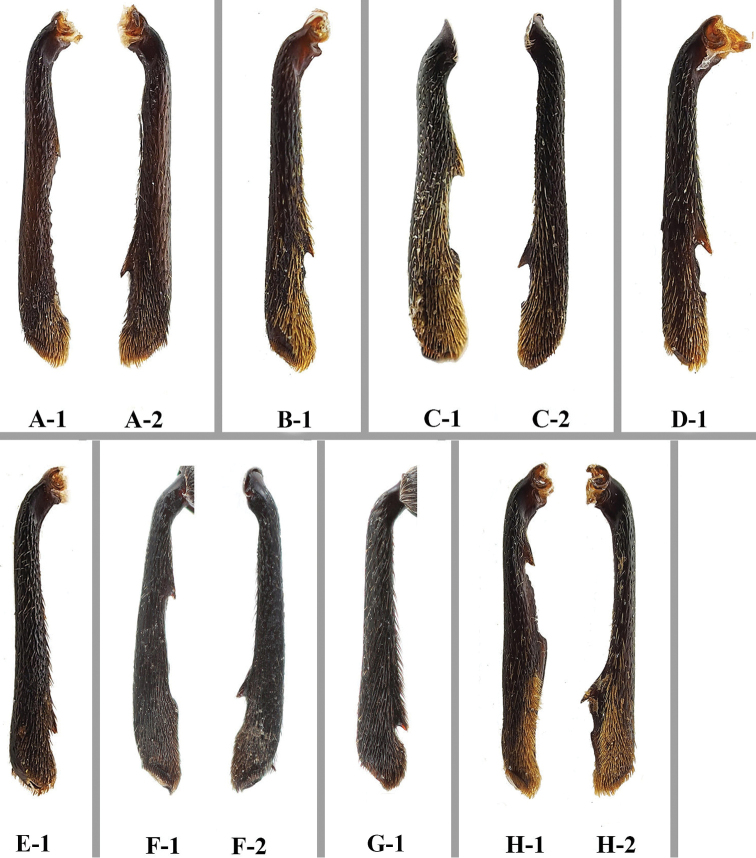
Male mesotibiae of *Sinocymbachus* spp **A***S. angustefasciatus***B***S. bimaculatus***C***S. decorus***D***S. excisipes***E***S. humerosus***F***S. luteomaculatus***G***S. parvimaculatus***H***S. quadrimaculatus*. **1** left **2** right.

**Figure 16.**
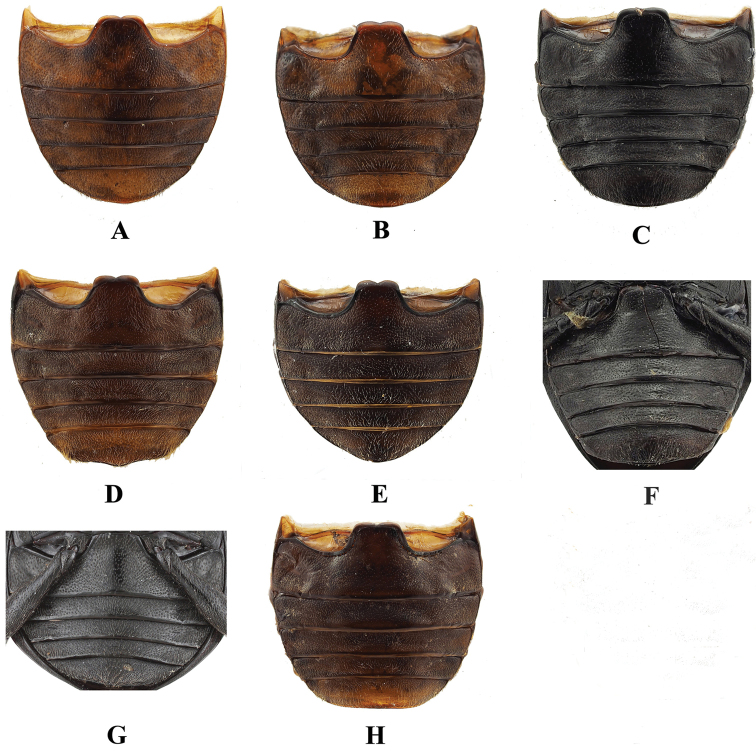
Male abdomen of *Sinocymbachus* spp **A***S. angustefasciatus***B***S. bimaculatus***C***S. decorus***D***S. excisipes***E***S. humerosus***F***S. luteomaculatus***G***S. parvimaculatus***H***S. quadrimaculatus*.

**Figure 17.**
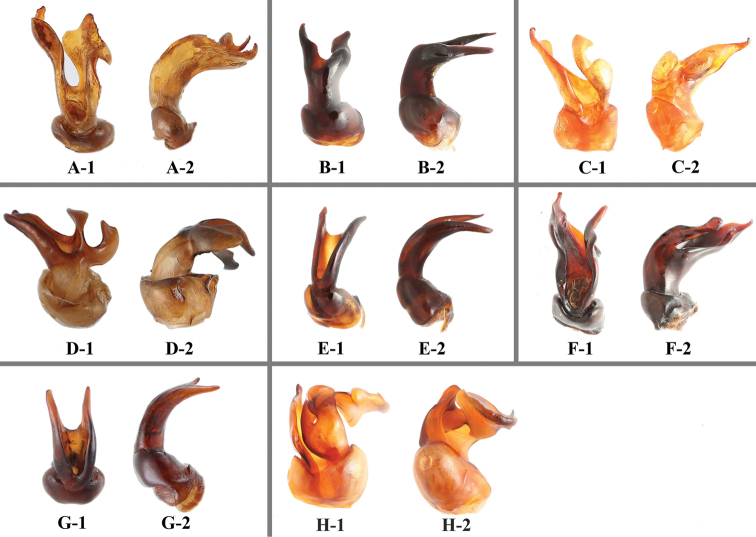
Aedeagus of *Sinocymbachus* spp **A***S. angustefasciatus***B***S. bimaculatus***C***S. decorus***D***S. excisipes***E***S. humerosus***F***S. luteomaculatus***G***S. parvimaculatus***H***S. quadrimaculatus*. 1 ventral view 2 lateral view.

**Figure 18.**
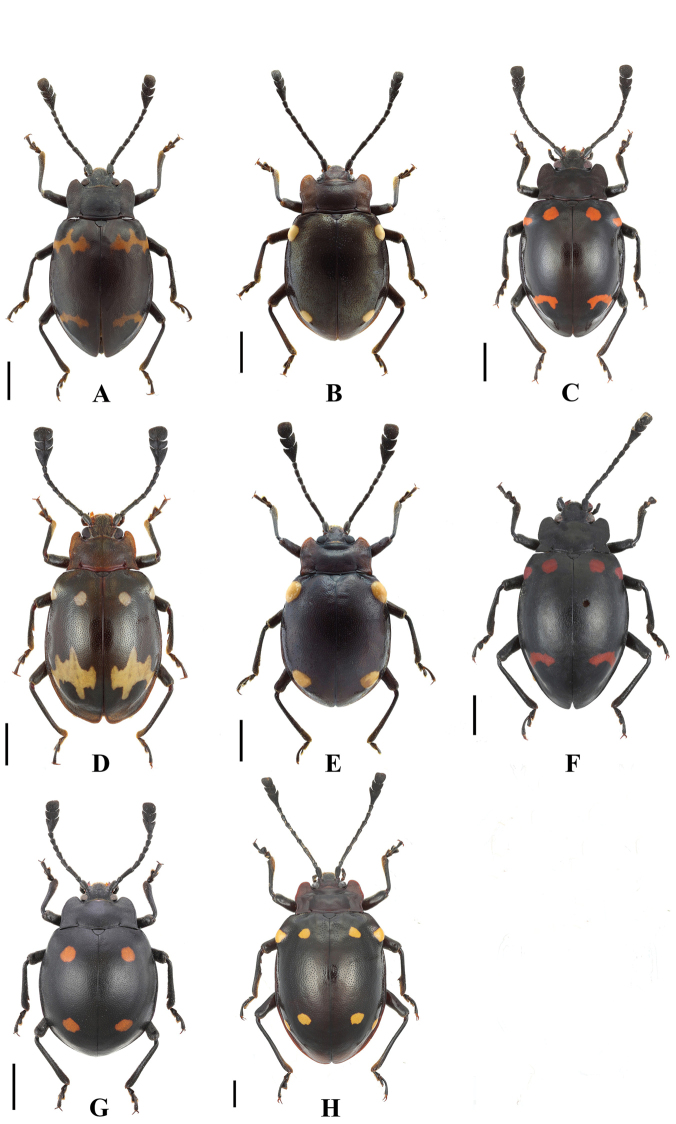
Habitus of *Sinocymbachus* spp. (males) **A***S. angustefasciatus***B***S. bimaculatus***C***S. decorus***D***S. excisipes***E***S. humerosus***F***S. luteomaculatus***G***S. parvimaculatus***H***S. quadrimaculatus*. Scale bar: 2 mm.

### Sinocymbachus quadriundulatus

Taxon classificationAnimaliaColeopteraEndomychidae

(Chûjô, 1938)

D450B7EE-4680-534D-9FD1-A99F3DAD42D5

Amphisternus quadriundulatus Chûjô, 1938: 397.
Sinocymbachus quadriundulatus : Strohecker and Chûjô 1970: 515.

#### Diagnosis.

Based on Strohecker and Chûjô (1970), *S. quadriundulatus* close to *S. angustefasciatus*, *S. fanjingshanensis* sp. nov., *S. longipennis* sp. nov. and *S. wangyinjiei* sp. nov. by the similar elytral patterns. But can be readily distinguished from them by the mesotibiae of male with symmetric tooth.

#### Length.

7.5–8.5 mm.

#### Distribution.

China: Taiwan.

### Key to the species of *Sinocymbachus* (modified and updated from Strohecker and Chûjô 1970)

**Table:** 

1	Elytra very short, nearly circular	**2**
–	Elytra oval or short oval	**3**
2	Each elytron with four spots; elytral sides strongly converging from 1/2 length of elytron to apex	***S. koreanus* comb. nov.**
–	Each elytron with two spots; elytral sides gradually converging from 1/2 length of elytron to apex (Fig. 18G)	***S. parvimaculatus***
3	Intercoxal process of mesoventrite with large tubercle at base (Fig. 14D); ventrite 5 with posterior margin abruptly projecting medially in male (Fig. 16D)	***S. excisipes***
–	Intercoxal process of mesoventrite with short median ridge at base; ventrite 5 with posterior margin not projecting medially in both sexes	**4**
4	Apical elytral maculae composed of round spots	**5**
–	Apical elytral macula transverse	**7**
5	Base of elytron with two spots (Fig. 18H)	***S. quadrimaculatus***
–	Base of elytron with one spot	**6**
6	Scutellum distinctly longer than wide (Fig. 7A); mesotibial tooth in male placed near apical 1/3 length of tibia (Fig. 15B–1)	***S. bimaculatus***
–	Scutellum as long as wide or slightly longer than wide (Fig. 7B); mesotibial tooth in male placed near apical 1/4 length of tibia (Fig. 15E–1)	***S. humerosus***
7	Basal elytral maculae composed of two spots	**8**
–	Basal elytral macula transverse	**10**
8	Body shiny; humeri distinctly prominent	**9**
–	Body opaque; humeri weakly prominent (Fig. 18F)	***S. luteomaculatus***
9	Body with cupreous sheen; basal elytral maculae arranged in horizontal line	***S. politus***
–	Body without cupreous sheen; basal elytral maculae arranged in oblique line (Fig. 18C)	***S. decorus***
10	Elytra short oval; elytral maculae without distinct projections (Fig. 1C)	***S. sinicus* sp. nov.**
–	Elytra oval or long oval, elytral maculae with distinct projections	**11**
11	Elytra distinctly long oval (especially in males); sides nearly parallel (Fig. 1B)	***S. longipennis* sp. nov.**
–	Elytra oval; sides curved	**12**
12	Body opaque (Fig. 18A)	***S. angustefasciatus***
–	Body shiny	**13**
13	Body with cupreous sheen; mesotibial tooth in male symmetric	***S. quadriundulatus***
–	Body without cupreous sheen; mesotibial tooth in male asymmetric	**14**
14	Anterior margin of mesoventral process as wide as posterior margin (Fig. 3A)	***S. fanjingshanensis* sp. nov.**
–	Anterior margin of mesoventral process much wider than posterior margin (Fig. 3D)	***S. wangyinjiei* sp. nov.**

## Supplementary Material

XML Treatment for Sinocymbachus

XML Treatment for Sinocymbachus fanjingshanensis

XML Treatment for Sinocymbachus longipennis

XML Treatment for Sinocymbachus sinicus

XML Treatment for Sinocymbachus wangyinjiei

XML Treatment for Sinocymbachus angustefasciatus

XML Treatment for Sinocymbachus bimaculatus

XML Treatment for Sinocymbachus decorus

XML Treatment for Sinocymbachus excisipes

XML Treatment for Sinocymbachus humerosus

XML Treatment for Sinocymbachus koreanus

XML Treatment for Sinocymbachus luteomaculatus

XML Treatment for Sinocymbachus parvimaculatus

XML Treatment for Sinocymbachus politus

XML Treatment for Sinocymbachus quadrimaculatus

XML Treatment for Sinocymbachus quadriundulatus

## References

[B1] Arriaga-Varela E Tomaszewska KW Navarrete-Heredia JL (2007) A synopsis of the Endomychidae (Coleoptera: Cucujoidea) of Mexico. Zootaxa 1594: 1–38.

[B2] Boo HJ (2013) Taxonomy of Endomychidae Leach (Coleoptera: Cucujoidea) in Korea. Korean Journal of Applied Entomology 53(1): 39–49. 10.5656/KSAE.2013.09.0.046

[B3] Bousquet Y (2004) The works of P.F.M.A. Dejean, with emphasis on publication dates and new carabid taxa proposed. Fabreries 29: 33–48.

[B4] Chûjô M (1938) Some additions and revisions to the Japanese Endomychidae (Coleoptera). Transactions of the Natural History Society of Formosa 28: 394–406.

[B5] Chûjô M Lee CE (1993) Endomychidae from Korea (Insecta, Coleoptera). Esakia 33: 95–98.

[B6] Mader L (1938) Neue Coleopteren aus China und Japan nebst Notizen. Entomologische Nachrichtenblatt (Troppau) 12: 40–61.

[B7] Pic M (1921) Nouveautés diverses. Mélanges Exotico-Entomologiques 34: 1–33.

[B8] Pic M (1927) Coléoptères de l’Indochine. Mélanges Exotico-Entomologiques 49: 1–36.

[B9] Pic M (1940) Diagnoses de Coléoptères exotiques. L’Échange, Revue Linnéenne 56: 10–12.

[B10] Robertson JA Ślipiński SA Moulton M Shockley FW Giorgi JA Lord NP Mckenna DD Tomaszewska KW Forrester J Miller KB Whiting MF Mchugh JV (2015) Phylogeny and classification of Cucujoidea and the recognition of a new superfamily Coccinelloidea (Coleoptera: Cucujiformia). Systematic Entomology 1–34. 10.1111/syen.12138

[B11] Shockley FW Tomaszewska KW McHugh JV (2009) An annotated checklist of the handsome fungus beetles of the world (Coleoptera: Cucujoidea: Endomychidae). Zootaxa 1999: 1–113. 10.11646/zootaxa.1999.1.1

[B12] Strohecker HF (1943) Some fungus beetles of the family Endomychidae in the United States National Museum, mostly from Latin America and the Philippine Islands. Proceedings of the U.S. National Museum 93: 381–392. 10.5479/si.00963801.93-3168.381

[B13] Strohecker HF (1953) Coleoptera, Endomychidae. In: Wytsman P (Ed.) Genera Insectorum.Louis Desmet-Verteneuil, Bruxelles, 1–145.

[B14] Strohecker HF Chûjô M (1970) *Sinocymbachus*, new gen. from the Orient (Coleoptera: Endomychidae). Pacific Insects 12(3): 511–518.

[B15] Tomaszewska KW (2000) Morphology, phylogeny and classification of adult Endomychidae (Coleoptera: Cucujoidea). Annales Zoologici 50(4): 449–558.

[B16] Tomaszewska KW (2005) Phylogeny and generic classification of the subfamily Lycoperdininae with a reanalysis of the family Endomychidae (Coleoptera: Cucujoidea). Annales Zoologici 55 (suppl. 1): 1–172.

